# Case Report: Clinical response of ensartinib for inflammatory myofibroblastic tumor of the urinary bladder with multiple metastases and TPM4-ALK fusion

**DOI:** 10.3389/fonc.2025.1481602

**Published:** 2025-05-02

**Authors:** Hongtao Ren, Qi Cheng, Xi Chen, Dianjing Sui, Zhiyi Zhang, Fei Chen

**Affiliations:** ^1^ Four Department of Oncology, Jilin Cancer Hospital, Changchun, Jilin, China; ^2^ Department of Integrated TCM & Western Medicine, Jilin Cancer Hospital, Changchun, Jilin, China; ^3^ Department of Radiology, Jilin Cancer Hospital, Changchun, Jilin, China

**Keywords:** ensartinib, inflammatory myofibroblastic tumor, urinary bladder, multiple metastases, TPM4-ALK

## Abstract

**Background:**

Inflammatory myofibroblastic tumor of the urinary bladder (IMTUB) is a rare tumor with low postoperative recurrence and metastasis. Due to the lack of clinical evidence, the optimal treatment paradigm for patients with IMTUB has not yet been established.

**Case presentation:**

We reported a case of a 55-year-old man who was diagnosed with bladder malignancy after transurethral resection of a bladder tumor, and then tumor metastasis was treated by traditional Chinese medicine. Following further disease progression, he was admitted to our hospital, where the diagnosis was revised to IMTUB with multiple metastases and TPM4–anaplastic lymphoma kinase (ALK) fusion by computed tomography (CT) scan, pathological diagnosis, immunohistochemistry, and genetic testing. The patient subsequently received 225 mg ensartinib once daily. Symptoms improved and achieved partial response (PR) with acceptable toxicities.

**Conclusion:**

Ensartinib may provide a new therapeutic direction with promising efficacy and an acceptable safety profile for IMTUB with ALK fusion. Further clinical investigation is needed to identify its efficacy and safety.

## 1 Introduction

Inflammatory myofibroblastic tumor (IMT) is a distinctive moderate malignant mesenchymal tumor with a global incidence of 0.04%–0.7% (1). In addition, IMT affects a variety of organs but is highly rare in the urinary bladder, where anaplastic lymphoma kinase (ALK) fusion is present in approximately 50% of cases (2, 3). Surgical resection is the standard treatment strategy of IMT of the urinary bladder (IMTUB), and the prognosis is relatively good (4). Only a few patients have postoperative recurrence and metastasis, and their treatment options are relatively limited (5, 6).

Ensartinib is a novel, potent, and highly selective next-generation ALK inhibitor that exhibits a broader inhibitory profile and demonstrates potent antitumor activity with favorable safety (7, 8). Furthermore, it has been shown to effectively inhibit ALK fusions in certain cancers (8, 9). Currently, there are no reports on treating IMTUB with ensartinib. Here, we reported the treatment results of ensartinib in a patient with multiple metastases and *TPM4-ALK* fusion of primary IMTUB, symptomatic improvement, and rapid decline in tumor burden with partial response (PR). Our patient was reported to provide new insight and evidence for the diagnosis and treatment of IMTUB.

## 2 Case presentation

A 55-year-old man initially presented with painless gross hematuria and received transurethral resection of a bladder tumor, subsequently considered as bladder malignancy by pathological examination (the specific record was unknown) at a Japanese hospital. Then, positron emission tomography (PET)–computed tomography (CT) revealed bladder tumor metastasis in our hospital and received Chinese medicine treatment (the specific prescription was unknown) at a local Chinese medicine clinic. However, a CT performed at Jilin People’s Hospital suggested further deterioration.

**Figure 1:**
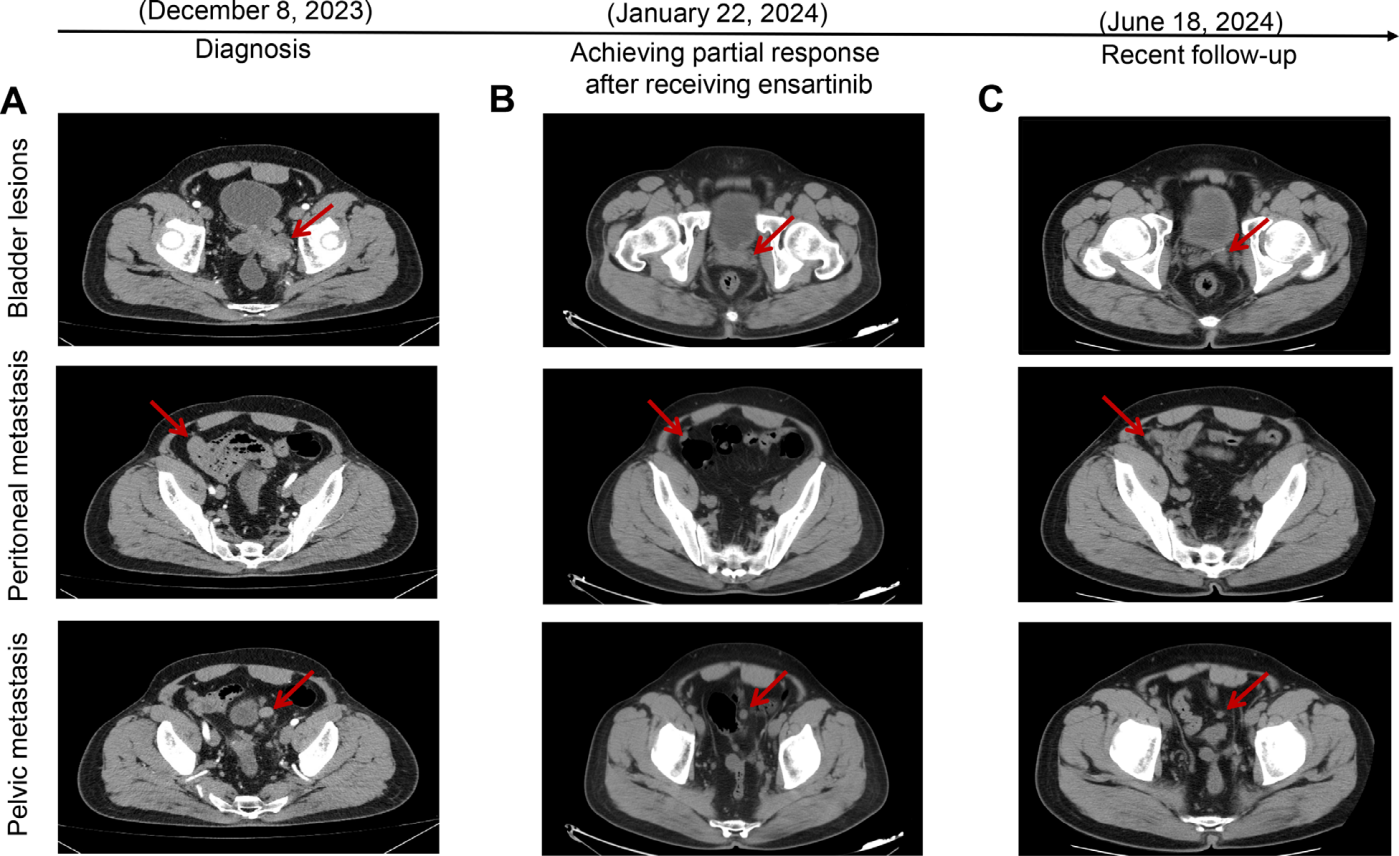
Target lesions of the patient with IMTUB by CT scans. **(A)** CT scans indicated space-occupying lesions of the bladder, with peritoneal and pelvic metastasis. **(B)** CT scans revealed tumors had decreased in size, achieving partial response after receiving ensartinib treatment. **(C)** Recent follow-up CT scans showed that the patient maintained partial response. IMTUB, inflammatory myofibroblastic tumor of the urinary bladder; CT, computed tomography.

**Figure 2:**
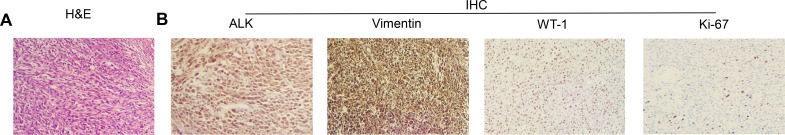
Morphologic features and IHC staining of the IMTUB samples. **(A)** Representative pictures of H&E staining (×100). **(B)** IHC analysis of ALK, Vimentin, WT-1, and Ki-67 expression levels (×100). H&E, hematoxylin and eosin; IHC, immunohistochemistry; IMTUB, inflammatory myofibroblastic tumor of the urinary bladder.

Upon admission to our department, the patient underwent a thorough evaluation, which included imaging, histopathological analysis, immunohistochemical studies, and next-generation sequencing (NGS). CT combined with magnetic resonance imaging (MRI) revealed space-occupying lesions of the bladder, considered to be a malignant tumor and recurrent, involving the left ureter, left seminal vesicle gland, and the anterior wall of the rectum (**Figure 1A**); pelvic lymph nodes were slightly larger than normal, and peritoneal thickening with multiple nodules led to the consideration of metastasis (**Figure 1A**); tiny nodules were present in the left upper lobe of the lung. Cystoscopy combined with histopathological examination revealed inflammatory cell infiltration of the urothelial mucosa and proliferating spindle cells present in the stroma, which was confirmed as a spindle cell tumor (**Figure 2A**). Immunohistochemistry (IHC) showed strong positive ALK (+) and Vimentin (+) staining, along with WT-1 (+), Ki-67 (approximately 20%+), CK (−), EMA (−), P40 (−), SMA (−), CD34 (+), Bcl-2 (focal weak +), S-100 (−), CD45 (−), PHH3 (+), CDla (−), and Desmin (−) (**Figure 2B**). NGS revealed three somatic cell variants, in which the representation mutation was *TPM4-ALK* Exon8:Exon20 fusion, with an abundance of 12.5%. Based on these findings, the patient was diagnosed with IMTUB, pelvic lymph node metastasis, peritoneal metastasis, and pulmonary nodules.

Given the presence of IMTUB with TPM4-ALK fusion and the treatment history, off-label treatment with ensartinib was considered, based on its mechanism of action as well as previous case reports demonstrating success with its use. The patient received oral ensartinib 225 mg once daily initiated on December 20, 2023. However, after approximately 1 month of treatment, the patient experienced swelling and a rash on the face, which were assessed by the investigator to be related to ensartinib and resolved soon with 40 mg/day furosemide tablets and 80 mg/day spironolactone tablets. Tumor evaluations were performed via CT, which revealed that both the bladder tumor and the metastatic sites had decreased in size compared to previous examinations on January 22, 2024 (**Figure 1**). According to Response Evaluation Criteria in Solid Tumors (RECIST) 1.1, the better response evaluation was evaluated as PR. The recent follow-up CT showed that the patient maintained PR for nearly 5 months (**Figure 1C**). The targeted therapy of ensartinib was continued, and regular outpatient follow-ups continued.

## 3 Discussion

IMTUB, a neoplasm of intermediate biologic potential with a local tumor recurrence rate of only 4% after surgery and just a few cases of metastasis to other organs, has been reported (6, 10). It presented challenges in treatment due to the rarity and complexity of its recurrence and metastasis, and conventional cytotoxic chemotherapy regimens seem to be ineffective (11, 12). In addition, the ALK fusion genes have been identified as tumor driver genes and potential therapeutic targets, and targeted therapy with ALK inhibitors, such as lorlatinib and alectinib, exhibited favorable antitumor activity in IMTUB with ALK fusion (13, 14). However, to our knowledge, this is the first case report describing the postoperative recurrence of IMTUB with multiple metastases and *TPM4-ALK* fusion treated with ensartinib.

The majority of IMTUB patients are young and female, with no obvious specificity, and the most common clinical manifestations are hematuria, frequency-dysuria syndrome, and bladder outlet obstruction (15). IMTUB radiographic examination usually indicates a space-occupying lesion in the bladder, which can be easily misdiagnosed as bladder malignancy due to tumor bleeding or surrounding blood clot accumulation. The definite diagnosis needs to rely on pathological and immunohistochemical results (16). Additionally, IMTUB is mostly a benign process, but a few cases have local invasion, recurrent, or malignant transformation (17, 18). Here, we described an extremely unusual case of recurrent IMTUB with multiple metastases, where the possible mechanism of recurrence was TPM N-terminal coiled-coil domains fused to the ALK C-terminal kinase domain, leading to abnormal proliferation and malignant transformation (19). In this context, ALK inhibition appears to be a necessary condition for preventing further deterioration.

The ALK inhibitor ensartinib is a promising treatment for ALK-positive non-small-cell lung cancer (NSCLC) and showed superior efficacy to crizotinib in systemic (20). However, unlike in NSCLC, evidence on the efficacy of ensartinib in IMT is limited. A 66-year-old man with IMT bone metastasis accompanied by *GCC2-ALK* fusion and another man with pulmonary IMT underwent postoperative disease progression with *STRN-ALK* fusion both obtained PR following treatment with ensartinib (21, 22). The *TPM4-ALK* fusion retains the complete kinase domain of ALK and has been identified in both pulmonary IMT and peritoneal IMT cases; however, treatment with entrectinib and crizotinib did not yield the expected therapeutic outcomes (23, 24). In addition, other novel ALK inhibitors, such as ceritinib, brigatinib, and alectinib, while proven to cause tumor shrinkage, inevitably lead to drug resistance or severe adverse reactions (14, 25). In this case, combined with previous treatment history and current condition, the patient adopted ensartinib therapy. After administration of the medication, the patient’s condition significantly improved, and he achieved PR, suggesting that the *TPM4-ALK* fusion gene may be a potential target for ensartinib in treating IMTUB. However, the specific efficacy of this treatment would need to be confirmed through large-scale clinical trials. Furthermore, regarding the adverse reactions, this patient experienced swelling and a rash on the face following the administration of ensartinib. Indeed, the rash and other skin toxic effects have been noted as the most frequently observed toxicities associated with ensartinib, as reported in various studies (20, 26). However, the mechanism behind ensartinib-induced skin toxicity remains unclear. Nevertheless, it has been reported that the concentration of ensartinib in the skin was 9.0× higher than in the plasma, potentially explaining the high frequency of skin-related side effects observed with its use (27). Importantly, the unique disease characteristics and genetic variations in each patient can greatly influence their response to the medication, thus necessitating the development of targeted treatment plans tailored to individual patients, potentially leading to greater clinical benefits for them.

There are some limitations in this case report. First, this case report involved experience with a single patient; it does not provide sufficient evidence to prove the efficacy of ensartinib in treating this population. Second, the inherent design of case reports may bias our findings and limit their generalizability. Finally, due to the limited research on ensartinib for the treatment of IMTUB, we lack comparative data to position our findings against other studies, so the findings of this case should be interpreted with caution.

## 4 Conclusion

In conclusion, the finding from this case suggests that *TPM4-ALK* may be used as a new target for IMTUB TKI treatment with ensartinib, which may provide a new therapeutic direction with promising efficacy and an acceptable safety profile. However, further clinical studies are needed to verify its benefits comprehensively.

## Data Availability

The original contributions presented in the study are included in the article/supplementary material. Further inquiries can be directed to the corresponding author.
